# Deep learning based on small sample dataset: prediction of dielectric properties of SrTiO_3_-type perovskite with doping modification

**DOI:** 10.1098/rsos.231464

**Published:** 2024-05-29

**Authors:** Quan Luo, Hua Hao, Hanxing Liu

**Affiliations:** ^1^ State Key Laboratory of Advanced Technology for Materials Synthesis and Processing, Center for Smart Materials and Device, International School of Materials Science and Engineering, Wuhan University of Technology, Wuhan, PR 430070, People's Republic of China

**Keywords:** deep learning, small dataset, perovskite structures, electronic constant, convolutional neural network, machine learning

## Abstract

The perovskite crystal structure represents a semiconductor material poised for widespread application, underpinned by attributes encompassing heightened efficiency, cost-effectiveness and remarkable flexibility. Notably, strontium titanate (SrTiO_3_)-type perovskite, a prototypical ferroelectric dielectric material, has emerged as a pre-eminent matrix material for enhancing the energy storage capacity of perovskite. Typically, the strategy involves augmenting its dielectric constant through doping to enhance energy storage density. However, SrTiO_3_ doping data are plagued by significant dispersion, and the small sample size poses a formidable research hurdle, hindering the investigation of dielectric property and energy storage density enhancements. This study endeavours to address this challenge, our foundation lies in the compilation of 200 experimental records related to SrTiO_3_-type perovskite doping, constituting a small dataset. Subsequently, an interactive framework harnesses deep neural network models and a one-dimensional convolutional neural network model to predict and scrutinize the dataset. Distinctively, the mole percentage of doping elements exclusively serves as input features, yielding significantly enhanced accuracy in dielectric performance prediction. Lastly, rigorous comparisons with traditional machine learning models, specifically gradient boosting regression, validate the superiority and reliability of deep learning models. This research advances a novel, effective methodology and offers a valuable reference for designing and optimizing perovskite energy storage materials.

## 1. Introduction

Perovskite materials have attracted widespread attention owing to their high photoluminescence quantum yield, high colour purity, tunable bandgap, wide colour gamut, high carrier mobility and long carrier diffusion length [1]. In the realm of complex oxide heterostructures with ABO_3_-type perovskite structures, notable physical phenomena have been observed. These phenomena encompass significant magnetoresistance, metal–insulator transitions, high-Tc superconductivity and two-dimensional electron gases. Strontium titanate (SrTiO_3_) stands as a quintessential inorganic compound with an ABO_3_-type perovskite structure. It serves as a widely used electronic functional ceramic material, boasting high dielectric constant, low dielectric loss and excellent thermal stability. It finds extensive applications in the realms of electronics, mechanics and ceramics industries [2]. SrTiO_3_ exhibits a broad bandgap (3.4 eV), excellent photocatalytic activity and distinct electromagnetic properties, along with redox catalytic activity. Consequently, it has found extensive use in photocatalytic domains, including photocatalytic water splitting for hydrogen production and the degradation of organic pollutants and photochemical cells [3–7]. Doping is a commonly used method to modify the electronic structure and magnetism of SrTiO_3_, allowing for its functionalization as a semiconductor, ferroelectric and magnetic material [8–10].

However, the impact of doping on the dielectric properties and energy storage density of SrTiO_3_ is not yet clear and requires extensive experimental data and theoretical calculations to support it. With the rapid development of computers, various computational methods such as density functional theory [11,12], first-principles calculations [13], molecular dynamics [14] and finite element models [15] have been widely applied and used for high-throughput screening. Although there have been advances in algorithms and computing conditions, performing large-scale first-principles calculations is still very expensive. In the meantime, the abundance of accessible databases in materials science has made the use of data-driven machine learning (ML) methods possible, which are increasingly being used to bypass these calculations [16,17]. Examples of perovskite property prediction include band characteristics [18], optimal composition [19], dielectric performance [20], bandgap energy [21] and dielectric breakdown strength [22].

According to the choice of fitting algorithms, ML research is generally divided into two categories. The first category includes traditional ML methods [23], such as support vector regression, Gaussian process regression (GPR) and other non-neural network models. Stanev *et al*. [24] modelled the critical temperatures (Tc) of over 12 000 known superconductors available via the SuperCon database using several ML methods. Lin *et al*. [20] and Li *et al*. [25], respectively, predicted the polycrystalline dielectric constant and bandgap value of perovskite using traditional ML methods. All these models demonstrated extremely low prediction error with perfect predictive performance; however, a large amount of data in the datasets were based on publicly available databases calculated using mathematical models, which allowed the traditional ML models to have good fitting performance. In contrast, these models exhibited problems of low prediction accuracy, slow convergence speed and poor generalization ability when dealing with experimental data. This is because, in the field of perovskites and materials, research funding for experiments is expensive, leading to dispersed and sparse experimental data, small sample sizes and difficulties collecting and unifying datasets, which severely obstructs the study of the relationships between perovskite composition, structure and properties.

**Figure 1.**
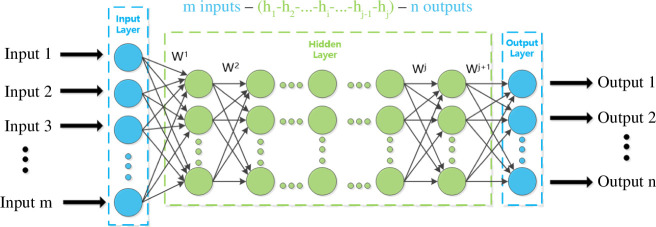
DNN model architecture diagram. The DNN model consists of an input layer, hidden layers and an output layer, with circles representing nodes in each layer. DNN, deep neural network.

At this time, the proposal of neural networks with strong data fitting and feature extraction capabilities led researchers to explore deep neural networks (DNNs) with many hidden layers that could be trained like the human nervous system, which is another type of ML method—deep learning (DL). The DNN model is a ML model based on an artificial neural network, as shown in figure 1, where circles represent neurons, arrows represent connections between neurons, W*^j^* represents the weight matrix of the *j*th layer and the number h*_i_* in parentheses represents the number of neurons in the *i*th layer. To deal with the difficulty of collecting experimental data, building small sample datasets and relying on reliable experimental data, neural networks can describe material properties in high-dimensional space as functions of composition and process parameters [26], effectively solving the shortcomings of traditional ML models, improving prediction accuracy and convergence speed and enhancing generalization ability. DNN has been widely applied in the field of materials science: in terms of predicting material properties, DNN can learn the relationship between material structure and properties to quickly and accurately predict physical, chemical, mechanical, electronic and other properties of materials [27,28]. For example, DNN can predict the elastic modulus of various types of materials such as metal alloys, ceramics and polymers [27], as well as the optoelectronic properties such as bandgap and carrier mobility of perovskite materials [28]; in terms of constructing phase diagrams, DNN can automatically construct phase diagrams of multi-component systems by learning the relationship between material stable phases and formation energies at different compositions and temperatures [29]. For example, DNN can construct phase diagrams of complex ternary systems such as aluminium–nickel–cobalt and Al–Ni–Zr [29] and verify them against experimental or first-principles computational results; in terms of material structure characterization, DNN can accelerate the design and characterization of material physical properties by learning the relationship between material structure features at different scales and target functions. For example, a DNN model can accelerate the design and characterization of the mechanical properties of non-uniform cellular materials by combining them with finite element analysis [30].

Although these methods have demonstrated that DNN can learn complex nonlinear mappings from large amounts of data and achieve or exceed human-level performance on multiple tasks, there are still very few studies on predicting small sample datasets in the field of materials science. This is because, unlike traditional ML models, DNN models cannot directly make predictions or evaluations by calling the model. The difficulty of training DNN lies in the fact that as the number of hidden layers (i.e. network depth) increases, gradients gradually disappear, leading to traps of poor local minima [31]. Feng *et al*. [26] and Yu *et al*. [32] both avoided local minima using stacked autoencoders (SAE) and fine-tuning, respectively, and achieved higher accuracy and smaller errors than traditional ML models in predicting the sensitivity of solidification cracking in material defects and the mechanical properties of aluminium alloys based on small sample datasets. However, the problem of parameter explosion in DNN remains unsolved. Since DNN adopts a fully connected form, the connections in the structure bring about an order of magnitude of weight parameters, which not only easily leads to overfitting but also easily causes traps in local optima. Moreover, pre-training and fine-tuning algorithms require high computer knowledge and typically require interdisciplinary researchers in both computer and materials science fields to conduct research, resulting in a waste of significant economic and time costs.

**Figure 2.**
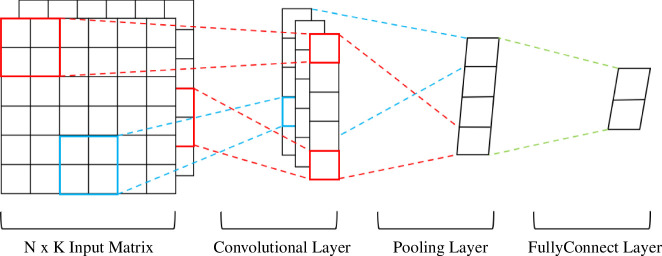
CNN model architecture diagram. The CNN model, in addition to the input and output layers, includes intermediate layers such as convolutional layers, pooling layers and fully connected layers. CNN, convolutional neural network.

Another neural network architecture belonging to the deep learning method is the convolutional neural network (CNN), which mainly improves the problem of parameter explosion in DNN. The network structure is shown in figure 2 [33], where not all upper and lower layer neurons are directly connected, but connected through ‘convolution kernels’ as intermediaries (partial connections). Owing to the characteristic of limiting the number of parameters and mining local structures, most research on CNN has focused on image recognition [34–37]. However, CNN’s role in data regression problems is also extremely powerful. Cao *et al*. [38] combined CNN and Long Short Term Memory (LSTM) models to achieve high-accuracy prediction of water plant operation data; Malek *et al*. [39] used one-dimensional CNN to extract features from spectral data, combined with Support Vector Machine (SVM) and GPR for accurate regression prediction; Kołodziej *et al*. [40] estimated heart rate variability using one-dimensional CNN and achieved higher performance accuracy than Multilayer Perceptron (MLP) and SVM. These applications have all demonstrated CNN’s strong data fitting and feature extraction capabilities. However, there are very few studies on data regression in the field of materials science. Therefore, constructing a CNN model to predict material properties can fill this gap and achieve improved prediction accuracy and convergence speed.

In this work, experiments were conducted to investigate the doping flexibility around SrTiO3 (STO), with Sr^2+^ ions being doped at A-sites by Ba^2+^, Ca^2+^, Pb^2+^, Bi^3+^, etc., and Ti^4+^ being doped at B-sites by Zr^4+^, Al^3+^, etc. [41–43]. Furthermore, some rare earth elements such as Nd^3+^, Sm^3+^, Pr^3+^ and Dy^3+^ have been shown to improve the performance of the STO system [44–48]. Reliable experimental data from literature and data collected by the research group on STO doping modification were used to construct a small dataset based on the STO perovskite system, which includes process parameters such as element molar ratio, sintering temperature, preparation method, as well as physical descriptors such as cell parameters, cell volume and microstructure [49]. CNN and DNN models were constructed to predict the dielectric properties of the STO doping system, achieving better prediction accuracy and generalization performance than traditional ML models (gradient boosting regression, GBR). Both CNN and DNN models demonstrated high predictive performance for the small dataset, providing a new modelling approach for studying the correlation between composition, structure and properties in STO data.

## 2. Methodology

The construction of traditional ML models, such as the GBR model discussed in this article, can be divided into the following five steps: (i) collecting a dataset containing the target attribute; (ii) generating a feature set based on prior knowledge and intuition to describe the characteristics of specific materials; (iii) identifying important features highly correlated with the target attribute through feature selection; (iv) evaluating candidate ML algorithms and selecting the best algorithm; and (v) testing the effectiveness of the model on new data outside the dataset by applying the model to it [23]. Unlike traditional ML models that mainly focus on feature selection for model prediction, the emphasis and difficulty in deep learning methods lies in constructing predictive models suitable for the dataset. Currently, popular deep learning models include CNNs, DNNs and recurrent neural networks (RNNs). Visual Geometry Group (VGG) [50], proposed by a research team at the University of Oxford, is among those that have achieved tremendous success in practical applications. It is characterized by its use of small convolution kernels, which increases the depth of the model and improves its accuracy. GoogleNet [51], proposed by a research team at Google, uses an inception module that allows the model to increase its depth and accuracy without adding parameters.

**Figure 3.**
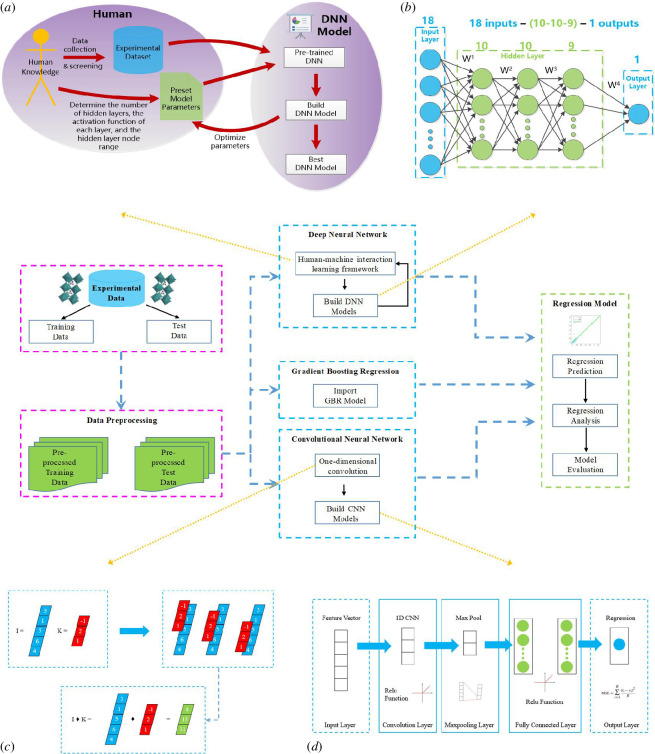
Overall framework of this study. This schematic introduces the details of data collection and partitioning, as well as the process of model construction and prediction, including GBR, DNN, CNN and regression analysis of results. (*a*) Human–machine interaction learning framework for building DNN models. Human-based theoretical models built on knowledge. Pre-trained models are used to adjust model parameters and the number of nodes in hidden layers to achieve ‘reconstructed’ models. The DNN model obtains the optimal structure for this small sample dataset through pre-training and reconstruction. (*b*) Example of a three-layer DNN model architecture. The number of nodes in the input layer, hidden layers and output layer are 18, 10, 10, 9 and 1, respectively. (*c*) One-dimensional valid convolution calculation process in the convolutional layer of the CNN model. (*d*) CNN model construction process. The model architecture mainly includes the input layer, convolutional layer (+ activation function), pooling layer, fully connected layer (+ activation function) and output layer (regression layer). CNN, convolutional neural network; DNN, deep neural network; GBR, gradient boosting regression.

In the present study, all construction steps can be divided into data collection, data preprocessing, building GBR, DNN and CNN models, prediction and regression analysis (as shown in figure 3). The GBR model used in this work was implemented in the Python programming language, which is commonly used in ML owing to being free and having numerous open-source packages that aid in ML, such as pandas [52], numpy [23], scipy [20] and scikit-learn [25]. The architectures of the DNN and CNN models were built using Matlab R2021a [53], which has a powerful deep learning toolbox [54] to assist researchers in constructing deep learning models suitable for their data, including mainstream models like VGG and GoogleNet.

### 2.1. Dataset

#### 2.1.1. Data collection

Vendik *et al*. [55,56] proposed analytical equations for calculating the complex dielectric constant of ferroelectric and paraelectric ferroelectric materials under different temperatures and electric fields. The equations are based on the traditional Landau theory, taking into account four energy dissipation mechanisms. Liu *et al*. [57] proposed a formula for calculating the complex dielectric constant of ceramic Ba_x_Sr_1-x_TiO_3_ with the recipe formula (2.1)


(2.1) $$\varepsilon \left(\text{E},T,f,x,\xi_{s}\right)=\frac{\varepsilon_{00}\left(x\right)}{G{\left(\text{E},T,x,\xi_{s}\right)}^{-1}+\sum_{q=1}^{4}\Gamma_{q}\left(\text{E},T,f,x,\xi_{s}\right)}.$$

In this formula, $G\left(\text{E},T,x,\xi_{s}\right)$ is the real part of the ferroelectric body’s dielectric response Green’s function, *x* represents the proportion of barium, *T* represents temperature and *f* represents the operating frequency of the bias field. $\varepsilon_{00}\left(x\right)$ is the simulation of the Curie–Weiss constant C, which can be expressed as $\varepsilon_{00}$ = C/Tc. $\xi_{s}$ is the statistical dispersion of the bias field (also known as the defect factor), which reflects the ‘quality’ of the material and corresponds to defects in the material (including oxygen vacancies and non-uniformities). Γ _1, 2, 3, 4_ represents the four energy dissipation (loss) mechanisms considered in the original model. Although this formula only applies to the BaTiO3 (BST) system, it also provides a reference for collecting data on the STO system. Starting from this formula, we established an experimental database of STO ceramic materials by selecting literature and research group experimental data. For non-data-oriented literature, if the data are displayed in graphical format rather than numerical format, we use WebPlotDigitizer [58] to extract the data from the graph.

The dielectric performance of ceramics refers to the polarization degree and loss in an electric field, which is influenced by factors such as material composition, structure and lattice defects of ceramics. The dielectric constant properties of SrTiO_3_ refer to its polarization degree in an electric field, which is influenced by factors such as temperature, frequency and doping, as evidenced by experimental data reported in the literature. This is why we chose the dielectric constant data of the STO system to establish our database. In the experimental testing of dielectric constants, different sample thicknesses and preparation process parameters are used, making it challenging to compare and draw consistent conclusions from data obtained from different studies. However, this is not an issue for DNNs. On the contrary, when we add these features to the dataset, the probability of CNNs, DNNs and other deep learning models discovering complex hidden relationships increases with the increase in data space variations.

In this study, a total of 200 sets of dielectric constant data for SrTiO_3_ doped and modified were collected from the literature and our research group. Unlike traditional ML approaches, the dataset used in this study is solely derived from experimental data, bypassing the complex and often resource-intensive feature extraction process typically required in conventional ML. This approach eliminates the need for extensive computational resources and time-consuming feature selection procedures. By using only the material compositions, specifically the stoichiometric ratios between the STO system and the dopants (Sr, Ti, O, Sm, Ba, Er, Hf, Zn, B, Si, Nd, Mn, Zr, Bi, Mg, Ca, Al and Sn), transformed into unified molar ratios represented in mol%, this study achieves remarkable predictive performance within the framework of deep learning models.

#### 2.1.2. Data screening and processing

**Figure 4.**
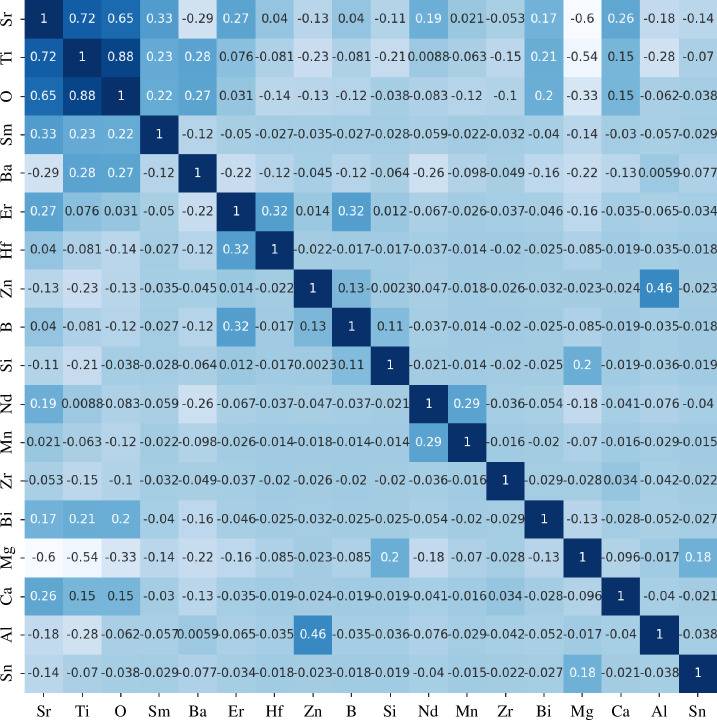
Pearson correlation coefficient diagram of the 18 variables in the dataset.


By using the Pearson correlation coefficient to screen the features in the dataset, assuming two variables *X* and *Y*, we have: in formula (2.2), $\bar{X}$ and $\bar{Y}$ are the means of *X* and *Y*, respectively. The Pearson correlation coefficients calculated range from −1 to 1, where a value close to 1 indicates a positive correlation between the two variables, a value close to −1 indicates a negative correlation and a value closer to 0 indicates less correlation between the two variables. Features with high correlation are removed, while those with low Pearson correlation coefficients are retained as inputs for ML. As shown in figure 4, when considering elements in the STO system, it was found that the Pearson correlation coefficients of Sr, Ti and O elements with the dopant element molar ratio had low correlation, and were, therefore, all retained as feature inputs for ML.

(2.2) $$Pearson=\frac{cov\left(X,Y\right)}{\sigma_{X}\sigma_{Y}}=\frac{\sum_{i=1}^{n}\left(X_{i}-\bar{X}\right)\left(Y_{i}-\bar{Y}\right)}{\sqrt{\sum_{i=1}^{n}{\left(X_{i}-\bar{X}\right)}^{2}\sum_{i=1}^{n}{\left(Y_{i}-\bar{Y}\right)}^{2}}}.$$

Unlike previous literature that mainly focused on ‘good’ data, which refer to data obtained by improving the dielectric properties of the STO system through doping modification, this study collected a portion of data that failed to improve or even decrease the dielectric constant of the STO system. These so-called ‘bad’ data, which are not suitable for traditional ML models for prediction, were also collected into the database and used to train the model. In summary, this measurement database comprises 16 distinct doping elements, totalling 200 data points (see electronic supplementary material), serving as the doping dataset for the STO system. As emphasized in this work, most of the data in this database represent STO composites; however, there is also a part that represents other components such as BST.

#### 2.1.3. Data analysis

**Figure 5.**
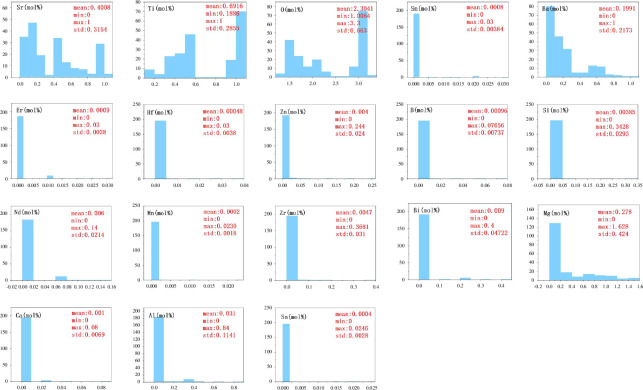
Data analysis of the dataset. Histograms of the 18 variables in the final dataset (sample size: 200), with statistical information such as mean, minimum, maximum and standard deviation displayed on the histograms.

The histogram in figure 5 displays the minimum, maximum and average values of 18 variables in the final dataset. Most of the variables, except for Sr, Ti, O, Ba and Mg elements, do not have a good distribution and are not suitable for modelling. However, with the powerful data fitting and feature extraction capabilities of deep learning models, the connections among these variables can be discovered, and good regression performance can be achieved. Feature selection was conducted based on the Pearson correlation coefficient, as shown in figure 4. Highly correlated features were discarded, and data with excessive missing parameters were deleted, resulting in a final dataset size of 200.

### 2.2. Data preprocessing

#### 2.2.1. Data normalization

To conform to ML input requirements, this study converted the collected mass ratios of STO and doping materials into molar ratios and normalized the data. Normalization is an essential preprocessing step in ML that can help the model converge faster. In this work, all features were normalized before the training process. The Matlab function mapminmax can be used to normalize the data for preprocessing by adjusting the parameters, and it can also be used to perform inverse normalization for regression prediction. Formula (2.3) shows the principle formula of this function for normalization processing, where *X* represents the input data to be processed, *X*_max_ and *X*_min_ are the maximum and minimum values, respectively, and *Y* represents the normalized output matrix. The values of *Y*_max_ and *Y*_min_ are generally set to 1 and −1, respectively.


(2.3) $$Y=\frac{\left(Y_{max}-Y_{min}\right)*\left(X-X_{min}\right)}{\left(X_{max}-X_{min}\right)}+Y_{min}.$$

#### 2.2.2. Division of dataset

To achieve an unbiased division of training and test data, we employed a stratified sampling approach. Initially, all data were grouped, and within each group, random selections were made for inclusion in the test set. This resulted in a random 9:1 split for selecting training and test data, using 9/10 of the dataset (180 data points) for training. The remaining 1/10 of the data (20 data points) was excluded to assess the generalization performance of the trained neural network, specifically its prediction accuracy for unseen data.

It is important to note that during the training process, as depicted in figure 3*a* of the human–machine interaction framework for constructing the DNN, the training set data were further divided into training set 1 and validation set in a 7:2 ratio. This division was also applied during the training of the CNN, ensuring the precision of the constructed DNN and CNN models.

### 2.3. Deep neural networks

#### 2.3.1. Framework for constructing deep neural networks

Figure 1 shows the model architecture of the DNN, which generally consists of an input layer, hidden layers and an output layer. While both the input and output layers consist of only one layer, the number of hidden layers is generally not fixed. The relationship between the number of hidden layers and the performance of the DNN model is shown in table 1 [31].

**Table 1.** Relationship between the number of hidden layers in the DNN model and results.

number of hidden layers	result
none	only capable of representing linear separable functions or decisions
1	can approximate any function that contains a continuous mapping from one finite space to another
2	can represent an arbitrary decision boundary to arbitrary accuracy with rational activation functions and can approximate any smooth mapping to any accuracy
>2	additional layers can learn complex representations (sort of automatic feature engineering) for layer layers

In theory, deeper hidden layers should enhance the fitting ability of the model and yield better results. However, in practice, too many hidden layers may lead to overfitting, increase the training difficulty, and make the model difficult to converge. Therefore, when using BP neural networks, it is best to refer to existing models with excellent performance. If there are no such models, start with one or two hidden layers, as shown in the table, and try to avoid using too many layers. One should not disregard practical considerations and blindly stack multiple neural networks together. It is also helpful to use transfer learning and fine-tuning [23] of pre-trained models, which can result in significant performance improvements. In material science regression problems, some published models have shown excellent performance with a DNN structure consisting of four hidden layers (21-(6-5-4-3)-1) for predicting solidification cracking susceptibility (SCS) [26], while others have used a three-hidden-layer DNN structure (14-(24-16-8)-1) for predicting the mechanical properties of aluminium alloys [32]. Based on this study, we established the best DNN model with 1–5 hidden layers and compared the prediction accuracy of different numbers of hidden layers.

Although the DNN model has shown strong generalization ability for defects and properties of these materials, it also indicates that the number of hidden layers and the number of nodes in each layer of the DNN model are not fixed when dealing with different small datasets. Currently, there is no scientific or universal method for determining the number of hidden layer nodes. The basic principle of selecting the number of hidden layer nodes is to use a compact structure with as few hidden nodes as possible while meeting the accuracy requirements. It is found that the number of hidden nodes is not only related to the number of nodes in the input/output layer but also to the complexity of the problem to be solved, the type of activation function used in the transformation and the characteristics of sample data. The number of nodes in the hidden layer is generally less than *N*-1, where *N* is the number of training samples.

However, since the DNN model requires the determination of a large number of parameters, it also incurs significant time and economic costs. Therefore, the real challenge is to develop effective DNN models that can fit small-sample experimental data well. In this work, we proposed a human–machine interactive learning framework, as shown in figure 3*a*. Based on the theoretical model (2.1) established by humans on the knowledge of the STO doping system, we collected and screened experimental datasets. We initialized the model parameters such as the number of hidden layers, the activation function of each layer and the number of nodes per layer using existing models or literature experience. We pre-trained the DNN model according to the dataset and initialized parameters set by human knowledge, and adjusted the model parameters based on the training results, including the number of hidden layer nodes, to achieve model ‘reconstruction’. As shown in the framework of figure 3*a*, we learned and established the DNN model, obtained the optimal parameters through pre-training, and reconstructed the DNN to obtain the best DNN model structure for predicting and regression analysis of this small dataset.

The DNN construction method provided in this study, based on training data, is precisely the method used to determine the number of hidden layers and corresponding nodes. The number of nodes per hidden layer is determined using the empirical formula (2.4).


(2.4) $$\text{HiddenNumber =}\sqrt{m+n}+a,$$

where *m* is the number of input layer nodes, *n* is the number of output layer nodes and *a* is generally an integer between 1 and 10. The range of hidden layer numbers is from 1 to 5, and the activation functions are, respectively, *Y* = tansig(*x*), *Y* = purelin(*x*), and *Y* = logsig(*x*), ReLU (*y* = max (*0*, *x*)). Our proposed framework is accepted by the inherent learning abilities of DL algorithms, which excel at quickly learning from large amounts of data, while humans use their analytical knowledge to abstract different domains to predict new situations, capturing even the slightest changes.

#### 2.3.2. Construct deep neural network model

Based on the human–computer interaction framework shown in figure 3*a*, a DNN model is constructed. The DNN model is initialized with 1–5 hidden layers, and a three-layer hidden layer architecture is used as an example, as shown in figure 3*b*. The pre-training phase involves training the model for 1000 epochs with a learning rate of 0.01 and a target minimum error of 0.000001. The mean squared error (MSE) on the validation set is calculated as the prediction accuracy during the pre-training phase, and it is used to determine the optimal number of nodes in the hidden layers. Based on the optimal number of hidden layers and nodes, DNN models with 1–5 hidden layers are established and trained again with the same network parameters as the pre-training phase. The trained models are then used for simulation, and the predicted results are denormalized. The denormalized results are compared with the true values of the testing set, and the calculated error values are used as the prediction error of the model.

### 2.4. Convolutional neural network

#### 2.4.1. Framework for constructing convolutional neural network

Figure 2 shows the structure and operating principle of CNN, which generally includes an input layer, convolutional layer, pooling layer, fully connected layer and output layer. The output layer is the regression layer for regression prediction. The steps for constructing a CNN model are shown in figure 3*d*. After preprocessing, data are input into the input layer and then processed by the convolutional layer and activation function. In this study, we used one-dimensional valid convolution as an example with a tensor I of length 5 and a kernel K of length 3. I is the input data matrix, and K is the convolution kernel. K moves along I sequentially, and at each fixed position, the corresponding values are multiplied and summed. Valid convolution only considers the case where K can completely cover I, that is K moves internally within I, as shown in figure 3*c*.

After the data are processed by the convolution operation, they are processed by the activation function before entering the pooling layer. The so-called activation function is a nonlinear mapping of the output results of the convolutional layer. If no activation function is used (which is equivalent to *f*(*x*) = *x*), in this case, the output of each layer is a linear function of the input of the previous layer. It can be concluded that no matter how many neural network layers there are, the output is a linear combination of the input, which is the same as the effect of no hidden layer, which is the most primitive perceptron.

Commonly used activation functions include the Sigmoid function (*y* = 1/(1+e^−*x*^)), Tanh function (*y* = (e^*x*^−e^−*x*^)/(e^*x*^+e^−*x*^)), ReLU (*y* = max (*0*, *x*)), etc. The activation functions used in this study are all ReLU.

Pooling, also known as under- or down-sampling, is mainly used for feature dimensionality reduction, compressing the number of data and parameters, reducing overfitting and improving the model’s fault tolerance. There are mainly two types: max pooling and average pooling, and we used the former in this study.

When the data reaches the fully connected layer, all neurons between the two layers are connected with weights, usually at the end of the CNN. That is, the connection method of the neuron is the same as that of the traditional neural network, as shown in figure 1, but the activation function needs to be added in the middle of the fully connected layer for processing.

Finally, the output layer performs regression prediction and calculates the MSE value of the model to obtain the final prediction result. The entire model training process is complete.

#### 2.4.2. Construct convolutional neural network model

The CNN model employed in this research comprises eight layers in its basic architecture, as illustrated in table 2. It includes an input layer, convolutional layers, pooling layers and an output layer, as well as two activation function layers and a fully connected layer. It uses the adaptive moment estimation (ADAM) algorithm as its optimization function. ADAM is characterized by its adaptability, fast convergence, low memory requirements and robustness, making it a widely adopted choice in the field of deep learning. The maximum number of training epochs is set at 1000, with a batch size of 24 for each iteration. The gradient threshold is set to 1, controlling the magnitude of gradients and truncating them when they exceed the threshold. The initial learning rate is set at 0.005.

**Table 2.** CNN model architecture and parameter settings.

no.	name of layer	parameters
1	input layer	200 samples are converted as input to Matlab
2	Conv_1	16 filters 3 × 1 convolutions with stride 1
3	Relu	ReLU
4	Maxpool	2 × 1 max pooling with stride 2 and padding 0
5	fc_1	1 fully connected layer
6	Relu	ReLU
7	fc_2	1 fully connected layer
8	regression output	mean squared error with response

### 2.5. Gradient boosting regression [59]

#### 2.5.1. Principles of the gradient boosting regression model

The GBR model improves the predictive accuracy of the final regression results by minimizing the algorithm generated during the training process. The final model is an optimized individual decision tree and a staged additive ensemble that infers *f*(*X*) → *E*. The target property of interest, denoted as ‘*E*’, is predicted using input data and features of the perovskite material, represented as ‘*X*’. The relationship between the input and output is modelled using a function, denoted as ‘*f*(*X*)’. ML aims to determine this function by using a well-defined set of labelled perovskite data (known as the training set) and using the trained model to predict the target property ‘*E*’ of new perovskite materials not included in the training set. Typically, the algorithm weights several weak ensembles for predicting *E*, all of which are obtained from separate training exercises. The ensemble can be represented in the form of equation (2.5).

Equation (2.6) expresses the optimal weight coefficients after minimization using the loss function, where *n* is the training time, *X* is the input data, *E* is the target energy property, *w*_*n*_ is the distribution weight vector, $f(X_{i},w_{n})$ is the regression function and $F_{n-1}(X_{i})$ is the current model. The loss function *L* uses either squared error or absolute error.


(2.5) $$F_{N}\left(X\right)=\sum_{n=1}^{N}f\left(X,w_{n}\right),$$

(2.6) $$w_{n}=arg\;\underset{w_{n}}{min}\sum_{i=1}^{N}L\left(E_{i},F_{n-1}\left(X_{i}\right)+f\left(X_{i},w_{n}\right)\right).$$

#### 2.5.2. Parameters for the gradient boosting regression model

The GBR model employed in this study underwent ‘hyperparameter optimization’ to tailor it for data regression prediction in small datasets. Among these parameters, the maximum number of iterations was set to 2000, and the learning rate, typically tuned in conjunction with the former, was set to 0.1 in this study. The decision tree’s maximum depth was set at 2, the minimum number of samples required for internal node splitting was 2 and the loss function used was MSE.

### 2.6. Prediction accuracy

For each ML model, three standard errors are used to assess the performance accuracy of the prediction exercise. They include the mean absolute error (MAE), the root mean square error (RMSE) and the *R*-square or coefficient of determination (*R* ^2^). The MAE equation (2.7) measures the average magnitude of errors in the set of predictions. The RMSE equation (2.8) has the benefit of penalizing large errors and is useful when large errors are particularly undesirable. The *R* ^2^ metric equation (2.9) is a statistical measure of how close the data fit to a regression line and is measured in percentage. Both the MAE and RMSE express average model predictions in the units of the variable and are negatively oriented scores, meaning the lower, the better [60].


(2.7) $$MAE=\frac{1}{n}\sum_{i=1}^{n}|E_{i}-E_{i}|,$$

(2.8) $$RMSE=\sqrt{\frac{1}{n}\sum_{i=1}^{n}{\left(E_{i}-E_{i}\right)}^{2}},$$

(2.9) $$RSE=\frac{\sum_{i=1}^{n}(E_{i}-E_{i}{)}^{2}}{\sum_{i=1}^{n}(E_{i}-\bar{E_{i}}{)}^{2}}.$$

$R^{2}=1-RSE$ , where *RSE* is the relative square error. Here, $\bar{E_{i}}$ denotes the predicted value of the variable *E*; and $\bar{E_{i}}$ is the mean value with summation over *n* number of samples, that is, i=1,2,...,n .

## 3. Results and discussion

### 3.1. Training GBR/DNN/CNN

In addition, this research also conducted training and testing of the GBR model to validate the accuracy advantage of DNN and CNN. The GBR model was trained and predicted using preprocessed input data, using Python language and the GBR model from the scikit-learn library.

**Figure 6.**
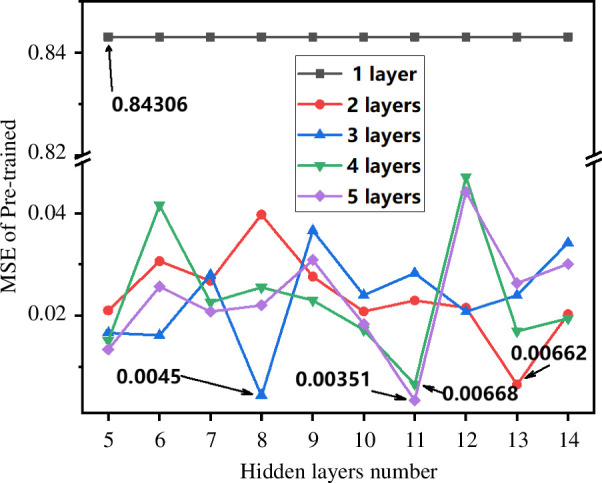
Model error during the pre-training phase of the original dataset as the number of nodes in hidden layers varies. The optimal nodes are 5, 13, 8, 11 and 11, with corresponding errors of 0.84306, 0.006623, 0.004504, 0.00668 and 0.003511.

The structure of the DNN is illustrated in figure 3*b*. It initially takes input from all elements, and the DNN thus constructed consists of 18 input neurons (each corresponding to one input variable), hidden neurons (with varying numbers) and one output neuron. The transfer function for the output layer is a linear function, purelin. The training algorithm used for the model is trainlm. During the pre-training phase, using the human–machine interaction framework proposed in this study (as depicted in figure 3*a*), the DNN with the following structure was trained: 18-(N1)-1, where N1, the number of nodes in the hidden layer, is determined based on empirical formula (2.2), ranging from 5 to 14. In the pre-training phase, other parameter values were set as follows: maximum training epochs (epoch) as 1000, learning rate as 0.01 and target minimum error as 0.000001. Figure 6 illustrates the variation of pre-training error with the increase in the number of nodes in the hidden layer, and it is observed that the optimal number of hidden nodes is 5, with a corresponding MSE of 0.84306. Thus, the final DNN structure with one hidden layer is determined as 18-(5)-1.

Similarly, DNNs with different configurations, including two hidden layers with 13 and 12 nodes, respectively, structured as 18-(13-12)-1, and DNNs with 3, 4 and 5 hidden layers structured as 18-(8-7-6)-1, 18-(11-10-10-9)-1 and 18-(11-10-10-9-9)-1, respectively, were trained using random initialization and pre-training techniques such as SAE and fine-tuning. The numbers inside the parentheses represent the neuron indices of the corresponding hidden layers. The optimal DNN models for the entire input dataset were confirmed through a human–computer interaction framework and pre-training, and they were found to be 18-(5)-1, 18-(13-12)-1, 18-(8-7-6)-1, 18-(11-10-10-9)-1 and 18-(11-10-10-9-9)-1.

CNNs were trained using error analysis on a validation set, and the lowest RMSE of 0.0695 was achieved after 1000 rounds of training. The trained models were then used for data regression prediction.

Multiple iterations of training with different random seeds were performed for each GBR and DNN configuration, exceeding 100 times, and the best-performing DNN, GBR and CNN models were selected for comparison. The GBR model used fivefold cross-validation to prevent overfitting, and data normalization was applied to DNN and CNN to prevent overfitting as well. The GBR model in this study was implemented using the scikit-learn library in Python, with data preprocessing and regression prediction. All computations of deep learning models were conducted in Matlab R2021a using the statistics and ML toolbox, neural network toolbox and regression learners. Owing to the small dataset and pre-training, the training time for DNN and CNN configurations on a personal computer ranged from a few seconds to several tens of seconds, significantly shorter than training deep learning models for image recognition, which requires multiple graphics processing units (GPUs) and many hours or even days. Finally, MAE, RMSE and *R* ^2^ values between the computed target values and the predicted values from GBR, DNN and CNN were used as evaluation metrics for model prediction accuracy.

### 3.2. Compare model prediction accuracy

#### 3.2.1. Original dataset

Using the established DNN and CNN models, as well as the GBR model, regression prediction was performed on the unseen dataset from the original dataset. The results are presented in table 3.

**Table 3.** Prediction accuracy and error comparison results of models on the original dataset.

model	*R* ^2^	MAE	RMSE
GBR	0.8648	500.6329	1144.180983
one-layer DNN	/	16063.514	16362.0312
two-layer DNN	0.82954	629.9841	1284.5107
three-layer DNN	0.86614	601.7341	1138.292
four-layer DNN	0.87515	605.093	1099.3045
five-layer DNN	0.84778	726.4575	1213.8326
CNN	0.84131	745.9119	1239.3661

CNN, convolutional neural network; DNN, deep neural network; MAE, mean absolute error; RMSE, root mean square error.

From the table, we can discern that the DNN model constructed using the human–machine interaction framework performs exceptionally well, particularly the DNN model with four hidden layers, which exhibits the best fit. It achieves an impressively high *R* ^2^ value of 0.87515, surpassing the GBR, a traditional ML model, which achieves an *R* ^2^ value of 0.8648. However, the one-layer DNN model demonstrates very poor predictive accuracy on the original dataset, rendering its *R* ^2^ value meaningless. Furthermore, the prediction results exhibit significant errors, with both MAE and RMSE values even surpassing those of the GBR model. This discrepancy can be attributed to the inherent complexity of the STO dataset’s data space, underscoring that a single hidden layer DNN model is unsuitable for data regression prediction in this dataset.

As the number of hidden layers increases, the model accuracy shows a trend of initially increasing and then decreasing, which is not a linear increase, similar to the trend observed with the number of hidden layer nodes. This finding is consistent with the results of other researchers. Firstly, when we increase the number of neurons in the hidden layer, the training accuracy of the neural network easily reaches high scores, but the testing accuracy has limitations. Secondly, blindly increasing the number of hidden layers does not linearly increase the prediction accuracy of the DNN model and may even increase the errors and decrease the accuracy. This is one of the main reasons why researchers explore higher testing accuracy of DNN, representing true learning ability (training accuracy can be understood as memory ability).

It is worth noting that the CNN model’s performance on the original dataset is not satisfactory, with lower predictive accuracy compared with the GBR model. Furthermore, in the predictions on the original dataset, although the *R* ^2^ values for all three models (excluding the one-layer DNN) are relatively high, their error values MAE and RMSE are both around 500 and 1000, indicating significant errors. This suggests that all models exhibit poor performance within the original dataset’s range. Additionally, the deep learning models, DNN and CNN, do not demonstrate better regression fitting capabilities for the original dataset compared with traditional ML methods commonly used in materials-related applications. This discrepancy can be attributed to the fact that the input features of this study’s dataset consist solely of the molar ratios of doping elements in STO, representing complete experimental data rather than the extensive computational material descriptors used in other literature. Moreover, the dataset contains a large amount of colossal dielectric constant data, which also contributes to the moderate performance of ML on this dataset.

**Figure 7.**
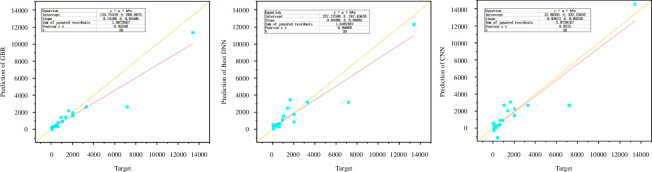
Prediction results of GBR, DNN and CNN models on unseen data in the original dataset. CNN, convolutional neural network; DNN, deep neural network; GBR, gradient boosting regression.

The predictions of the GBR model, the optimal DNN model and the CNN model on the original dataset are illustrated in figure 7. The horizontal axis represents the true values (‘Target’) of the original dataset, while the vertical axis represents the predictions of the three models (‘Prediction’). In the figure, the solid red line represents the linear regression equation. The closer the cyan data points are to the yellow dashed line, the more accurate the predictions. The table in the figure displays the equations, intercept, slope, sum of squared residuals, Pearson’s *r* value and sample size (*N*) corresponding to each type of image. However, in contrast to traditional ML models that rely solely on hyperparameter optimization, deep learning models, as demonstrated through the human–machine interaction framework proposed in this study, exhibit respectable predictive accuracy on the original dataset. Additionally, owing to their more intuitive model architecture, they show significant potential for performing data regression prediction on small datasets.

#### 3.2.2. Small dataset

**Figure 8.**
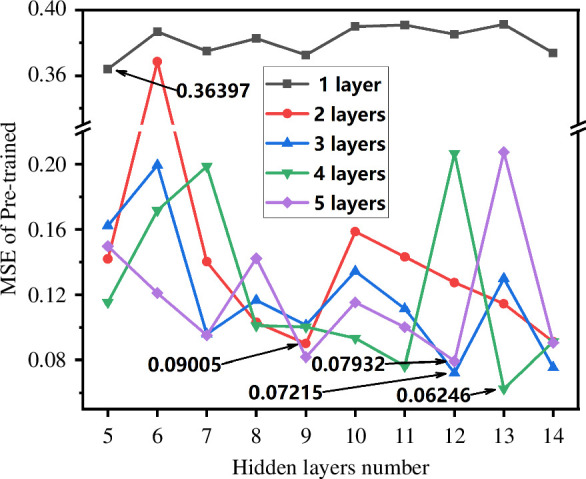
Model error during the pre-training phase of the small dataset as the number of nodes in hidden layers varies. The optimal nodes are 5, 9, 12, 13 and 12, with corresponding errors of 0.36397, 0.09005, 0.072151, 0.062461 and 0.079323.

To further validate the robust fitting and generalization ability of DNN and CNN on small-sample datasets, we conducted a reselection process on the small-sample dataset by removing the doping components related to the giant dielectric constant, particularly the Mn element, resulting in a new small-sample dataset with reduced input features to 17 and a dataset size of 125 samples. Meanwhile, the GBR and CNN model architectures used in this section remained unchanged, with the number of input features or nodes decreasing with the reduction of input features. However, the DNN model was reconstructed using the human–computer interaction framework shown in figure 3*a*, which further demonstrates the versatility and user-friendliness of the framework proposed in this study. The DNN model was first pre-trained on the small-sample dataset, and figure 8 shows the variation of pre-training error with the increase of hidden layer node number. From the figure, it can be observed that the optimal number of hidden layer nodes is 5, with a corresponding MSE of 0.36397. Therefore, the optimal model structure for DNN with 1 hidden layer is 17-(5)-1.

Similarly, DNN models with two hidden layers were trained with different numbers of hidden nodes, including 9 and 8, resulting in model structures of 17-(9-8)-1. DNN models with three, four and five hidden layers had model structures of 17-(12-11-10)-1, 17-(13-13-12-11)-1 and 17-(12-11-11-10-10)-1, respectively, where the numbers in parentheses represent the neuron numbers in the corresponding hidden layers. All DNN models were trained with random initialization and underwent pre-training and fine-tuning through SAE. After the human–computer interaction framework and pre-training, the optimal DNN models for the selected small dataset were determined to be 17-(5)-1, 17-(9-8)-1, 17-(12-11-10)-1, 17-(13-13-12-11)-1 and 17-(12-11-11-10-10)-1.

**Figure 9.**
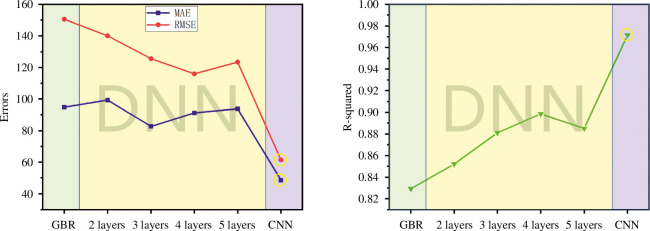
Comparison of prediction accuracy and error of GBR/DNN/CNN models on the small dataset. CNN, convolutional neural network; DNN, deep neural network; GBR, gradient boosting regression; MAE, mean absolute error; RMSE, root mean square error.

Finally, GBR, DNN and CNN models were retrained on the small-sample dataset, and the performance on the final unseen dataset was tested, as shown in figure 9. Compared with all the models used on the original dataset, the predictive model for the small dataset has eliminated the ineffective one-layer DNN model.

In figure 9, the green portion on the left represents the predictive accuracy of the GBR model. It is observed that the GBR model has a lower MAE compared with the two-layer DNN model but higher than the three-, four- and five-layer DNN models, significantly higher than CNN. The RMSE values are the highest among the three models, and the *R* ^2^ value is the lowest. Additionally, in contrast to the GBR model applied to the original dataset, the GBR model on the small dataset exhibits a performance decline. This suggests that the GBR model has relatively poorer data fitting capabilities on small datasets compared with deep learning models. However, it still demonstrates stable predictive performance when compared with the unoptimized or unprocessed DNN (one-layer DNN). This indirectly confirms the scientific validity of using traditional ML models for material property prediction in the field of materials. While achieving stable predictive performance is important, especially considering the extensive time and economic costs associated with material computations, achieving higher data fitting capabilities on small datasets requires the integration of deep learning models, which are indispensable in the field of computer science for significant applications.

The middle yellow section in figure 9 displays the changes in *R* ^2^ value, MAE and RMSE of the DNN model’s predictions on the small dataset as the number of hidden layers increases. The results indicate that as the number of hidden layers increases from two to four, the DNN model’s *R* ^2^ value rises from 0.85203 to its peak at 0.8986. However, as the number of hidden layers further increases to five, the *R* ^2^ value decreases to 0.88513. In the left graph, the patterns for RMSE and MAE are opposite. With an increase in the number of hidden layers from two to five, the error trends show a decrease followed by an increase. It is noteworthy that the RMSE reaches its minimum value with the four-layer hidden DNN model, while the MAE achieves its minimum with the three-layer DNN structure. However, the overall model accuracy is still not as high as that of the four-layer DNN model. In summary, the 17-(13-13-12-11)-1 structure in the DNN model exhibits the highest accuracy, which is consistent with the predictive results of the DNN model applied to the original dataset.

The right-hand side purple part of figure 9 represents the prediction accuracy and error of the CNN model. It can be observed that the CNN model has the smallest prediction errors in terms of MAE and RMSE among all the models and the highest *R* ^2^ value among all the models. This indicates that the CNN model has the best prediction performance for the small sample dataset of the STO system in this study. This not only proves that deep learning has a much better data fitting ability than traditional ML models but also demonstrates that the CNN model has a better data regression ability for small sample datasets in the field of materials science compared with the DNN model.

Table 4 shows the comparison of prediction accuracy and errors of the traditional ML model GBR, the best DNN model and the CNN model. Among them, the CNN model has the highest *R* ^2^ value, which is 0.9715, and the smallest prediction errors. The DNN model with the structure of 17-(13-13-12-11)-1, which contains four hidden layers, has the best accuracy with an *R* ^2^ value of 0.8986. Both deep learning models have higher testing accuracy than the GBR model, with improvements of 0.1423 and 0.0694, respectively. The prediction errors are significantly reduced compared with the GBR model, with the highest MAE value reduced by 46.2065 and the highest RMSE value reduced by 89.1024. Compared with the original dataset error results in table 3, the MAE and RMSE values of the four-layer DNN model are reduced from 605.093 to 91.2099 and from 1099.3045 to 115.9266, respectively. At the same time, the MAE and RMSE values of the CNN model’s prediction results are reduced from 745.9119 to 48.617 and from 1239.3661 to 61.3909, respectively. Its error reduction is quite remarkable, with the *R* ^2^ value increasing from 0.84131 to 0.9715. This also demonstrates the powerful data fitting and predictive performance of deep learning models on small datasets.

**Table 4.** Prediction accuracy and error comparison results of the GBR model, optimal DNN model, and CNN model on the small dataset.

model	*R* ^2^	MAE	RMSE
GBR	0.8292	94.8235	150.4933
four-layer DNN	0.8986	91.2099	115.9266
CNN	0.9715	48.617	61.3909

CNN, convolutional neural network; DNN, deep neural network; GBR, gradient boosting regression; MAE, mean absolute error; RMSE, root mean square error.

**Figure 10.**
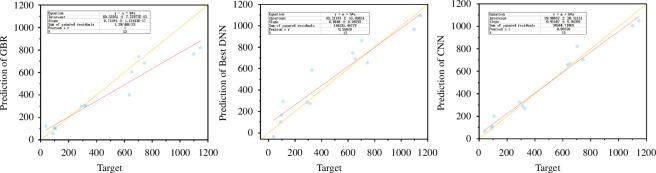
Prediction results of GBR, DNN and CNN models on unseen data in the small dataset. CNN, convolutional neural network; DNN, deep neural network; GBR, gradient boosting regression.

Figure 10 shows the linear regression plots of the target values versus the predicted values of GBR, the best DNN model and the CNN model on the unseen test dataset. In the figure, the horizontal axis labelled ‘Target’ represents the actual values of dielectric constants in the test dataset, while the vertical axis labelled ‘Prediction’ corresponds to the predicted values from the GBR, DNN and CNN models. In the figure, the solid red line represents the linear regression equation. The closer data points are to the yellow dashed line, the more accurate the predictions. As shown in the figure, both DNN and CNN models exhibit strong data regression capabilities on this small sample dataset, with a significant reduction in errors compared with the original dataset. Moreover, both deep learning models have higher accuracy than the GBR model.

## 4. Conclusions

In this study, we have established a small sample dataset of SrTiO_3_-based perovskite materials with modified dielectric constants through doping. This dataset includes ‘good’ and ‘bad’ data, and we have successfully predicted the energy storage performance using CNN and DNN models, improving performance prediction accuracy.

A vast array of scattered experimental data is succinctly quantified through deep learning regression to represent the influence of doping element molar ratios on target properties, with these ratios serving as inputs for deep learning. Compared with the common traditional ML methods applied in the field of materials science, deep learning regression bypasses the need for intricate feature computation, extraction and selection. It achieves higher predictive accuracy and generalization performance for small materials domain datasets by relying on straightforward material element ratios.

Although DNN and CNN are mostly used on large datasets, they are still highly accurate models when dealing with small datasets in the field of materials. Small datasets are common in materials research, therefore, deep learning models are more suitable than traditional ML models for solving materials-related problems, such as the study of perovskite doping modification on room temperature dielectric constant in this paper. Constructing deep learning models for small datasets using more suitable and efficient methods is effective and necessary. This study demonstrates that deep learning models constructed with small datasets and human–computer interaction frameworks have huge application prospects in materials research, particularly in dispersed, multi-variate nonlinear problems. In comparison with the DNN model used for the small dataset of the STO system, this study also demonstrates that the CNN model exhibits lower errors, superior data fitting and regression capabilities. This suggests a novel modelling approach for future research in the field of materials science, particularly for small-sample studies.

## Data Availability

The data used in this article and its references are provided in the electronic supplementary material [61].

## References

[1] Krieg F *et al* . 2018 Colloidal CsPbX_3_ (X = Cl, Br, I) nanocrystals 2.0: zwitterionic capping ligands for improved durability and stability. ACS Energy Lett. **3** , 641–646. (10.1021/acsenergylett.8b00035) 29552638 PMC5848145

[2] Lim H , *et al* . 2021 Nature of the surface space charge layer on undoped SrTiO_3_ (001). J. Mater. Chem. C **9** , 13094–13102. (10.1039/D1TC03436G)

[3] Tokura Y , Kawasaki M , Nagaosa N . 2017 Emergent functions of quantum materials. Nature Phys. **13** , 1056–1068. (10.1038/nphys4274)

[4] Zhu J , Lee JW , Lee H , Xie L , Pan X , De Souza RA , Eom CB , Nonnenmann SS . 2019 Probing vacancy behavior across complex oxide heterointerfaces. Sci. Adv. **5** , eaau8467. (10.1126/sciadv.aau8467) 30801011 PMC6386560

[5] Reinle-Schmitt ML *et al* . 2012 Tunable conductivity threshold at polar oxide interfaces. Nat. Commun. **3** , 932. (10.1038/ncomms1936) 22760631

[6] Gozar A , Logvenov G , Kourkoutis LF , Bollinger AT , Giannuzzi LA , Muller DA , Bozovic I . 2008 High-temperature interface superconductivity between metallic and insulating copper oxides. Nature **455** , 782–785. (10.1038/nature07293) 18843365

[7] Ohtomo A , Hwang HY . 2004 A high-mobility electron gas at the LaAlO_3_/SrTiO_3_ heterointerface. Nature **427** , 423–426. (10.1038/nature02308) 14749825

[8] Muta H , Kurosaki K , Yamanaka S . 2003 Thermoelectric properties of rare earth doped SrTiO_3_ . J. Alloys Compd. **350** , 292–295. (10.1016/S0925-8388(02)00972-6)

[9] Janousch M , Meijer GI , Staub U , Delley B , Karg SF , Andreasson BP . 2007 Role of oxygen vacancies in Cr‐doped SrTiO_3_ for resistance‐change memory. Adv. Mater. **19** , 2232–2235. (10.1002/adma.200602915)

[10] Konta R , Ishii T , Kato H , Kudo A . 2004 Photocatalytic activities of noble metal ion doped SrTiO_3_ under visible light irradiation. J. Phys. Chem. B **108** , 8992–8995. (10.1021/jp049556p)

[11] Rizwan M , Ali A , Usman Z , Khalid NR , Jin HB , Cao CB . 2019 Structural, electronic and optical properties of copper-doped SrTiO_3_ perovskite: a DFT study. Physica B Condens. Matter **552** , 52–57. (10.1016/j.physb.2018.09.022)

[12] Wang Y , Lv Z , Zhou L , Chen X , Chen J , Zhou Y , Roy VAL , Han ST . 2018 Emerging perovskite materials for high density data storage and artificial synapses. J. Mater. Chem. C **6** , 1600–1617. (10.1039/C7TC05326F)

[13] Stengel M . 2011 First-principles modeling of electrostatically doped perovskite systems. Phys. Rev. Lett. **106** , 136803. (10.1103/PhysRevLett.106.136803) 21517406

[14] He R , et al . 2022 Structural phase transitions in SrTiO_3_ from deep potential molecular dynamics. Phys. Rev. B **105** , 064104. (10.1103/PhysRevB.105.064104)

[15] Yang S , Chen Y . 2015 Concurrent atomistic and continuum simulation of bi-crystal strontium titanate with tilt grain boundary. Proc. Math. Phys. Eng. Sci. **471** , 20140758. (10.1098/rspa.2014.0758) 25792957 PMC4353054

[16] Schlenz H , Baumann S , Meulenberg WA , Guillon O . 2022 The development of new perovskite-type oxygen transport membranes using machine learning. Crystals **12** , 947. (10.3390/cryst12070947)

[17] Ghosh A , Sumpter BG , Dyck O , Kalinin SV , Ziatdinov M . 2021 Ensemble learning-iterative training machine learning for uncertainty quantification and automated experiment in atom-resolved microscopy. npj Comput. Mater. **7** , 100. (10.1038/s41524-021-00569-7)

[18] Chen L , Xu B , Chen J , Bi K , Li C , Lu S , Hu G , Lin Y . 2020 Ensemble-machine-learning-based correlation analysis of internal and band characteristics of thermoelectric materials. J. Mater. Chem. C **8** , 13079–13089. (10.1039/D0TC02855J)

[19] Kang S , Park S , Kim KW , Woo SI , Park S . 2007 High-throughput screening of ferroelectric materials for non-volatile random access memory using multilayer perceptrons. Appl. Surf. Sci. **254** , 725–733. (10.1016/j.apsusc.2007.05.097)

[20] Lin X , Li C , Hao H , Zhao G , Liu H . 2021 Accelerated search for ABO_3_-type the electronic contribution of polycrystalline dielectric constants by machine learning. Comput. Mater. Sci. **193** , 110404. (10.1016/j.commatsci.2021.110404)

[21] Lu S , Zhou Q , Ouyang Y , Guo Y , Li Q , Wang J . 2018 Accelerated discovery of stable lead-free hybrid organic-inorganic perovskites via machine learning. Nat. Commun. **9** , 3405. (10.1038/s41467-018-05761-w) 30143621 PMC6109147

[22] Shen ZH , Wang JJ , Jiang JY , Huang SX , Lin YH , Nan CW , Chen LQ , Shen Y . 2019 Phase-field modeling and machine learning of electric-thermal-mechanical breakdown of polymer-based dielectrics. Nat. Commun. **10** , 1843. (10.1038/s41467-019-09874-8) 31015446 PMC6478924

[23] Li C , Hao H , Xu B , Zhao G , Chen L , Zhang S , Liu H . 2020 A progressive learning method for predicting the band gap of ABO_3_ perovskites using an instrumental variable. J. Mater. Chem. C **8** , 3127–3136. (10.1039/C9TC06632B)

[24] Stanev V , Oses C , Kusne AG , Rodriguez E , Paglione J , Curtarolo S , Takeuchi I . 2018 Machine learning modeling of superconducting critical temperature. npj Comput. Mater. **4** , 29. (10.1038/s41524-018-0085-8)

[25] Li C , Hao H , Xu B , Shen Z , Zhou E , Jiang D , Liu H . 2021 Improved physics-based structural descriptors of perovskite materials enable higher accuracy of machine learning. Comput. Mater. Sci. **198** , 110714. (10.1016/j.commatsci.2021.110714)

[26] Feng S , Zhou H , Dong H . 2019 Using deep neural network with small dataset to predict material defects. Mater. Des. **162** , 300–310. (10.1016/j.matdes.2018.11.060)

[27] Zhong X , Gallagher B , Liu S , Kailkhura B , Hiszpanski A , Han TYJ . 2022 Explainable machine learning in materials science. npj Comput. Mater. **8** , 204. (10.1038/s41524-022-00884-7)

[28] Nyshadham C , Rupp M , Bekker B , Shapeev AV , Mueller T , Rosenbrock CW , Csányi G , Wingate DW , Hart GLW . 2019 Machine-learned multi-system surrogate models for materials prediction. npj Comput. Mater. **5** , 51. (10.1038/s41524-019-0189-9)

[29] Feng S , Fu H , Zhou H , Wu Y , Lu Z , Dong H . 2021 A general and transferable deep learning framework for predicting phase formation in materials. npj Comput. Mater **7** , 10. (10.1038/s41524-020-00488-z)

[30] Ma C , Zhang Z , Luce B , Pusateri S , Xie B , Rafiei MH , Hu N . 2020 Accelerated design and characterization of non-uniform cellular materials via a machine-learning based framework. npj Comput. Mater. **6** , 40. (10.1038/s41524-020-0309-6)

[31] Goodfellow I , Bengio Y , Courville A . 2016 Deep learning. Cambridge, MA: MIT press.

[32] Yu Z , Ye S , Sun Y , Zhao H , Feng XQ . 2021 Deep learning method for predicting the mechanical properties of aluminum alloys with small data sets. Mater. Today Commun. **28** , 102570. (10.1016/j.mtcomm.2021.102570)

[33] Chen Y . 2015 Convolutional neural network for sentence classification. Master's thesis, University of Waterloo, Ontario, Canada.

[34] Zhang Z . 2023 Automated graph neural networks accelerate the screening of optoelectronic properties of metal-organic frameworks. J. Phys. Chem. Lett. **14** , 1239–1245. (10.1021/acs.jpclett.3c00187) 36716343

[35] Kattenborn T , Leitloff J , Schiefer F , Hinz S . 2021 Review on convolutional neural networks (CNN) in vegetation remote sensing. ISPRS J. Photogramm. Remote Sens. **173** , 24–49. (10.1016/j.isprsjprs.2020.12.010)

[36] Alzubaidi L *et al* . 2021 Review of deep learning: concepts, CNN architectures, challenges, applications, future directions. J. Big Data **8** , 53. (10.1186/s40537-021-00444-8) 33816053 PMC8010506

[37] Roska T , Chua LO . 1993 The CNN universal machine: an analogic array computer. IEEE Trans. Circuits Syst. II: Analog and Digital Signal Processing **40** , 163–173. (10.1109/82.222815)

[38] Cao K , Kim H , Hwang C , et al . 2018 CNN-LSTM coupled model for prediction of waterworks operation data. J. Inf. Process. Syst. **14** , 1508–1520. (10.3745/JIPS.02.0104)

[39] Malek S , Melgani F , Bazi Y . 2018 One‐dimensional convolutional neural networks for spectroscopic signal regression. J. Chemom. **32** , e2977. (10.1002/cem.2977)

[40] Kołodziej M , Majkowski A , Tarnowski P , Rak RJ , Rysz A . 2021 A new method of cardiac sympathetic index estimation using a 1D-convolutional neural network. B. Pol. Acad. Sci-Tech **69** , 136921. (10.24425/bpasts.2021.136921)

[41] Zhang B *et al* . 2015 High thermoelectric performance of Nb-doped SrTiO_3_ bulk materials with different doping levels. J. Mater. Chem. C **3** , 11406–11411. (10.1039/C5TC02016F)

[42] Kumar P . 2011 Dielectric and impedance properties of Sr(Sm0.5Nb0.5)O3 ceramics.. Solid State Sciences **13** , 2060–2065. (10.1016/j.solidstatesciences.2011.09.011)

[43] Tai B , Jin Y , Jinfeng W , Fadong P . 2022 Grain size engineered (Ba,Sr)(Zr,Ti)O_3_ ceramics with excellent energy storage properties for high-voltage pulsed capacitors. Ceram. Int. **48** , 17 046–17 052. (10.1016/j.ceramint.2022.02.260)

[44] Shen ZY , Li YM , Luo WQ , Wang ZM , Gu XY , Liao RH . 2013 Structure and dielectric properties of Nd x Sr1−x TiO_3_ ceramics for energy storage application. J. Mater. Sci. Mater. Electron. **24** , 704–710. (10.1007/s10854-012-0798-2)

[45] Hu QG , Shen ZY , Li YM , Wang ZM , Luo WQ , Xie ZX . 2014 Enhanced energy storage properties of dysprosium doped strontium titanate ceramics. Ceram. Int. **40** , 2529–2534. (10.1016/j.ceramint.2013.07.126)

[46] Sluchinskaya IA , Lebedev AI , Erko A . 2012 Crystal structure, local structure, and defect structure of Pr-doped SrTiO_3_ . J. Appl. Phys. **112** , 024103. (10.1063/1.4737586)

[47] Shi J , Ye J , Ma L , Ouyang S , Jing D , Guo L . 2012 Site-selected doping of upconversion luminescent Er^3+^ into SrTiO_3_ for visible-light-driven photocatalytic H_2_ or O_2_ evolution. Chem. Eur. J. **18** , 7543–7551. (10.1002/chem.201102807) 22532311

[48] Shen ZY , Hu QG *et al* . 2013 Structure and dielectric properties of Re_0.02_Sr_0.97_TiO_3_ (Re = La,Sm,Gd,Er) ceramics for high‐voltage capacitor applications. J. Am. Ceram. Soc. **96** , 2551–2555. (10.1111/jace.12364)

[49] Ramprasad R , Batra R , Pilania G , Mannodi-Kanakkithodi A , Kim C . 2017 Machine learning in materials informatics: recent applications and prospects. npj Comput. Mater. **3** , 54. (10.1038/s41524-017-0056-5)

[50] Sengupta A , Ye Y , Wang R , Liu C , Roy K . 2019 Going deeper in spiking neural networks: VGG and residual architectures. Front. Neurosci. **13** , 95. (10.3389/fnins.2019.00095) 30899212 PMC6416793

[51] Ballester P , Araujo R . 2016 On the performance of GoogLeNet and AlexNet applied to sketches. Proc. AAAI Conf. Artif. Intell. **30** , 1124–1128. (10.1609/aaai.v30i1.10171)

[52] Zhang N , Paluri M , Ranzato M , Darrell T , Bourdev L . 2014 PANDA: pose aligned networks for deep attribute modeling. Proc. of the IEEE Conf. on Computer Vision and Pattern Recognition 1637–1644. (10.1109/CVPR.2014.212)

[53] Mathworks . 2012 Matlab. Natick, MA: Mathworks.

[54] Paluszek M , et al . 2020 MATLAB machine learning Toolboxes. In Practical MATLAB deep learning: A project-based approach, pp. 25–41. Berkeley, CA: Apress. (10.1007/978-1-4842-5124-9)

[55] Vendik OG , Zubko SP . 1997 Modeling the dielectric response of incipient ferroelectrics. J. Appl. Phys. **82** , 4475–4483. (10.1063/1.366180)

[56] Vendik OG , Zubko SP , Nikol’ski MA . 2002 Microwave loss-factor of Ba_x_Sr_1−x_TiO_3_ as a function of temperature, biasing field, barium concentration, and frequency. J. Appl. Phys. **92** , 7448–7452. (10.1063/1.1524314)

[57] Liu N , Ihalage A , Zhang H , Giddens H , Yan H , Hao Y . 2020 Interactive human–machine learning framework for modelling of ferroelectric–dielectric composites. J. Mater. Chem. C **8** , 10352–10361. (10.1039/C9TC06073A)

[58] Rohatgi A . 2019 WebPlotDigitizer – extract data from plots, images, and maps. See https://automeris.io/WebPlotDigitizer

[59] Friedman JH . 2001 Greedy function approximation: a gradient boosting machine. Ann. Statist. **29** , 1189–1232. (10.1214/aos/1013203451)

[60] Willmott CJ , Matsuura K . 2005 Advantages of the mean absolute error (MAE) over the root mean square error (RMSE) in assessing average model performance. Clim. Res. **30** , 79–82. (10.3354/cr030079)

[61] Luo Q , Hao H , Liu H . 2024 Supplementary material from deep learning based on small sample dataset: prediction of dielectric properties of SrTiO_3_-type perovskite with doping modification. FigShare (10.6084/m9.figshare.c.7158515) PMC11285775 39076810

